# Targeted insertion of an anti-CD2 monoclonal antibody transgene into the *GGTA1* locus in pigs using *Fok*I-dCas9

**DOI:** 10.1038/s41598-017-09030-6

**Published:** 2017-08-16

**Authors:** Mark B. Nottle, Evelyn J. Salvaris, Nella Fisicaro, Stephen McIlfatrick, Ivan Vassiliev, Wayne J. Hawthorne, Philip J. O’Connell, Jamie L. Brady, Andrew M. Lew, Peter J. Cowan

**Affiliations:** 1 Robinson Research Institute & Adelaide School of Medicine, University of Adelaide, Adelaide, Australia; 2 Immunology Research Centre, St. Vincent’s Hospital Melbourne, Melbourne, Victoria, Australia; 3 Westmead Millennium Institute, University of Sydney, Sydney, Australia; 4 Walter and Eliza Hall Institute, Melbourne, Victoria, Australia; 5 Department of Microbiology & Immunology, University of Melbourne, Victoria, Australia; 6 Department of Medicine, University of Melbourne, Victoria, Australia

## Abstract

Xenotransplantation from pigs has been advocated as a solution to the perennial shortage of donated human organs and tissues. CRISPR/Cas9 has facilitated the silencing of genes in donor pigs that contribute to xenograft rejection. However, the generation of modified pigs using second-generation nucleases with much lower off-target mutation rates than Cas9, such as *Fok*I-dCas9, has not been reported. Furthermore, there have been no reports on the use of CRISPR to knock protective transgenes into detrimental porcine genes. In this study, we used *Fok*I-dCas9 with two guide RNAs to integrate a 7.1 kilobase pair transgene into exon 9 of the *GGTA1* gene in porcine fetal fibroblasts. The modified cells lacked expression of the αGal xenoantigen, and secreted an anti-CD2 monoclonal antibody encoded by the transgene. PCR and sequencing revealed precise integration of the transgene into one allele of *GGTA1*, and a small deletion in the second allele. The cells were used for somatic cell nuclear transfer to generate healthy male knock-in piglets, which did not express αGal and which contained anti-CD2 in their serum. We have therefore developed a versatile high-fidelity system for knocking transgenes into the pig genome for xenotransplantation purposes.

## Introduction

Transplantation is an important treatment for a number of medical conditions, but the supply of human donor material remains a crucial limiting factor. Indeed, the widening gap between waiting list and transplant recipient numbers is driving research into the development of alternative sources including pigs (xenotransplantation). Genetic modification has long been used in an attempt to increase the compatibility of porcine organs and tissues with human recipients. Knockout (KO) of the *GGTA1* (α1,3-galactosyltransferase) gene, which is responsible for expression of αGal (the major xenoantigen that elicits hyperacute xenograft rejection) protects porcine xenografts in pig-to-nonhuman primate (NHP) models^1^, as does transgenic expression of human regulators of complement^2^ and coagulation^3^. However, long-term xenograft survival in the absence of ongoing immunosuppression remains difficult to achieve. To address this and other issues, many additional genetic modifications are currently under investigation. We have focused recently on engineering porcine cells to secrete immunomodulatory molecules to provide ‘local’ graft-specific immune protection, with the aim of reducing or eliminating the requirement for chronic systemic immunosuppression (and consequently its adverse effects). We generated an anti-CD2 monoclonal antibody (mAb) named diliximab that binds to T cells from humans, Old World primates and New World primates^4^. As proof of concept, we demonstrated that adenovirus-transduced porcine islets expressing this mAb were protected from infiltration by human T cells in a humanized mouse model^5^. However, transgenic pigs with this modification have not yet been produced.

One of the challenges in xenotransplantation has been the practical difficulty of combining multiple genetic modifications, including knockouts and randomly integrated transgenes, in a single donor pig. Recently, however, the development of gene editing technology has radically improved the capacity to efficiently modify the pig genome. Notably, CRISPR/Cas9 has been used to generate multi-KO pigs in a single step^6, 7^. A potential disadvantage of the wild type (WT) Cas9 nuclease is its propensity to introduce off-target mutations^8^; this produces an unknown threat and emotional burden that may be long term. To overcome this problem, newer versions of Cas9 with higher fidelity have been developed^9^. *Fok*I-dCas9, which consists of a nuclease-dead Cas9 fused to a subunit of the non-specific endonuclease *Fok*I, uses two guide RNAs to direct cleavage between appropriately spaced 20-nucleotide target sites, and has remarkably higher (>140 fold) specificity than WT Cas9^10^. A similar chimeric nuclease, fdCas9, shows on-target efficiency approaching that of WT Cas9 and an absence of activity at known WT Cas9 off-target sites^11^. *Fok*I-dCas9 has been used to generate KO mice^12^, but its use in pigs has not been reported. In addition, the demonstrated utility of the CRISPR system to generate knock-in pigs^13–15^ (cf. knockout pigs) has not yet been applied for xenotransplantation. To address these gaps, we established a *Fok*I-dCas9-based protocol to knock-in a potentially protective transgene, encoding the anti-CD2 mAb diliximab, into the *GGTA1* locus of pigs. This “kills two birds with one stone” by eliminating the gene associated with hyperacute rejection while introducing an immunomodulatory molecule.

## Results

### Targeting of *GGTA1* in porcine cells using *Fok*I-dCas9

**Figure 1:**
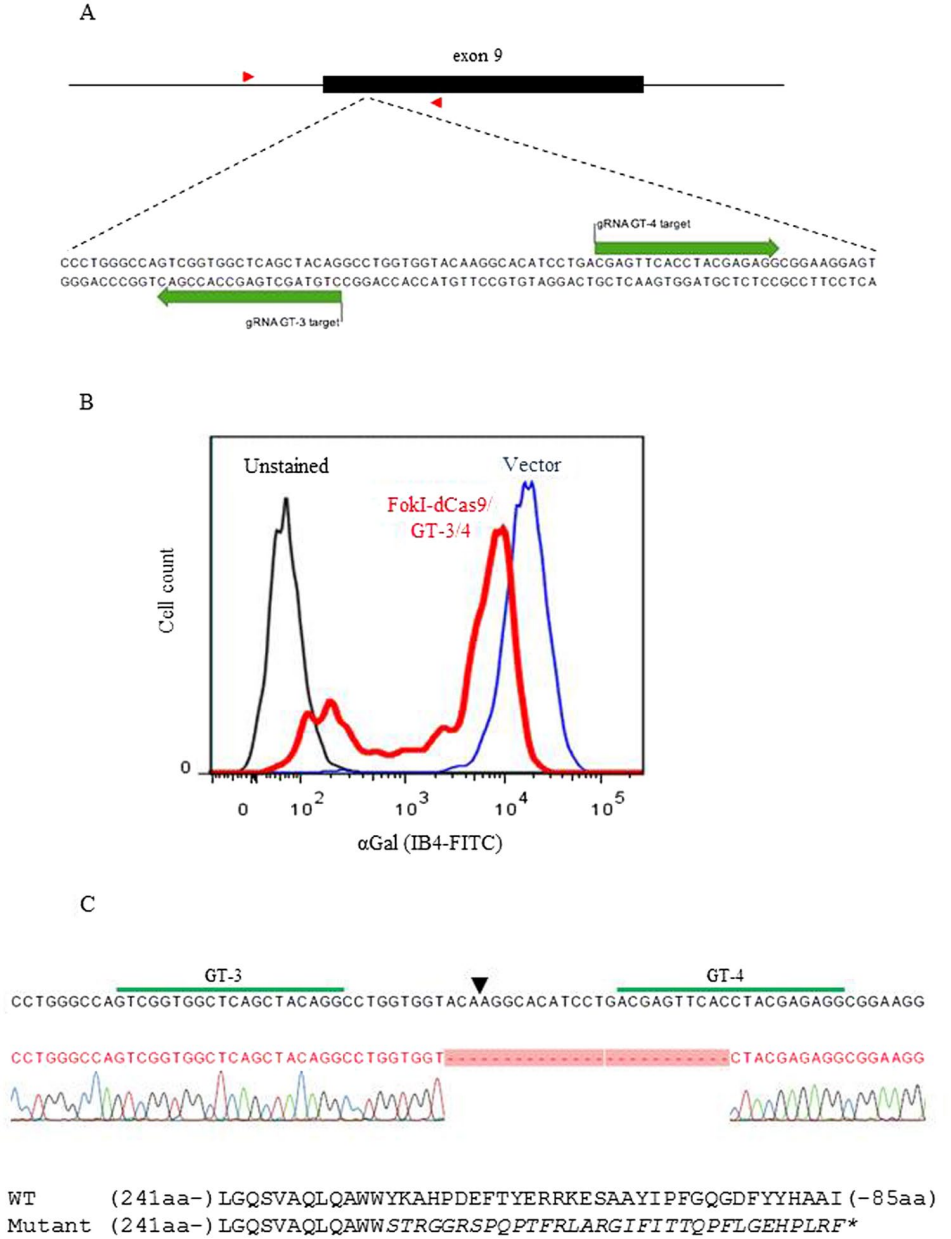
Targeting of *GGTA1* in WT pig fetal fibroblasts using *Fok*I-dCas9. (**A**) Target region within *GGTA1* exon 9. Green arrows indicate target sites for guide RNAs GT-3 and GT-4. Red arrowheads indicate binding sites for primers GTFS-F1 and GTFS-R1, used to amplify the target region after genome editing. (**B**) Flow cytometric analysis of αGal expression (IB4-FITC) on fibroblasts, three days after co-transfection with expression vectors for *Fok*I-dCas9, GT-3 and GT-4. A substantial proportion of the co-transfected cells (red line) showed a marked reduction in αGal expression compared to control vector-transfected cells (blue line); black line, unstained WT fibroblasts. (**C**) Sequence analysis of the target region, amplified from genomic DNA isolated from the pool of co-transfected cells. One clone contained a 25 bp deletion around the predicted cleavage site (arrowhead). The predicted amino acid sequences of WT and mutated α1,3-galactosyltransferase are shown below the sequence, indicating an altered stretch of 33 residues (italics) and truncation of 85 residues in the mutant protein.

We targeted exon 9 of *GGTA1* because it encodes the catalytic domain of α1,3-galactosyltransferase. A pair of guide RNAs (GT-3 and GT-4) was designed to recognize sites separated by 26 bp (Fig. 1A), centered on a region that has been shown to be accessible to gene editing^16^. The spacing was based on the observation that *Fok*I-dCas9 cleaves optimally with spacer lengths of ~15 or ~25 bp^10^. To test correct targeting, WT pig fetal fibroblasts were co-transfected with expression vectors for *Fok*I-dCas9, GT-3 and GT-4. Three days after transfection, the cells were harvested, stained for αGal expression, and analyzed by flow cytometry. Approximately 20% of the cells were negative for αGal (Fig. 1B), indicating biallelic knockout of *GGTA1*. By comparison, approximately 30% αGal-negative cells were obtained using WT Cas9 with a single guide RNA targeted to the same region (Suppl. Fig. 1 and Suppl. Table 1). The target region, amplified by PCR of genomic DNA isolated from the unsorted cells, showed fragments of the expected size when analyzed using the Surveyor nuclease assay (Suppl. Fig. 2). The amplified target region from the unsorted cells was ligated into a cloning vector, and sequencing of one randomly selected clone revealed a 25 bp deletion around the expected cleavage site, predicting a significant mutation of the catalytic domain of the α1,3-galactosyltransferase protein (Fig. 1C). Together these data demonstrate efficient targeting and cleavage of *GGTA1* exon 9 by *Fok*I-dCas9 guided by GT-3 and GT-4.

### Preparation and validation of an anti-CD2 mAb knock-in construct

**Figure 2:**
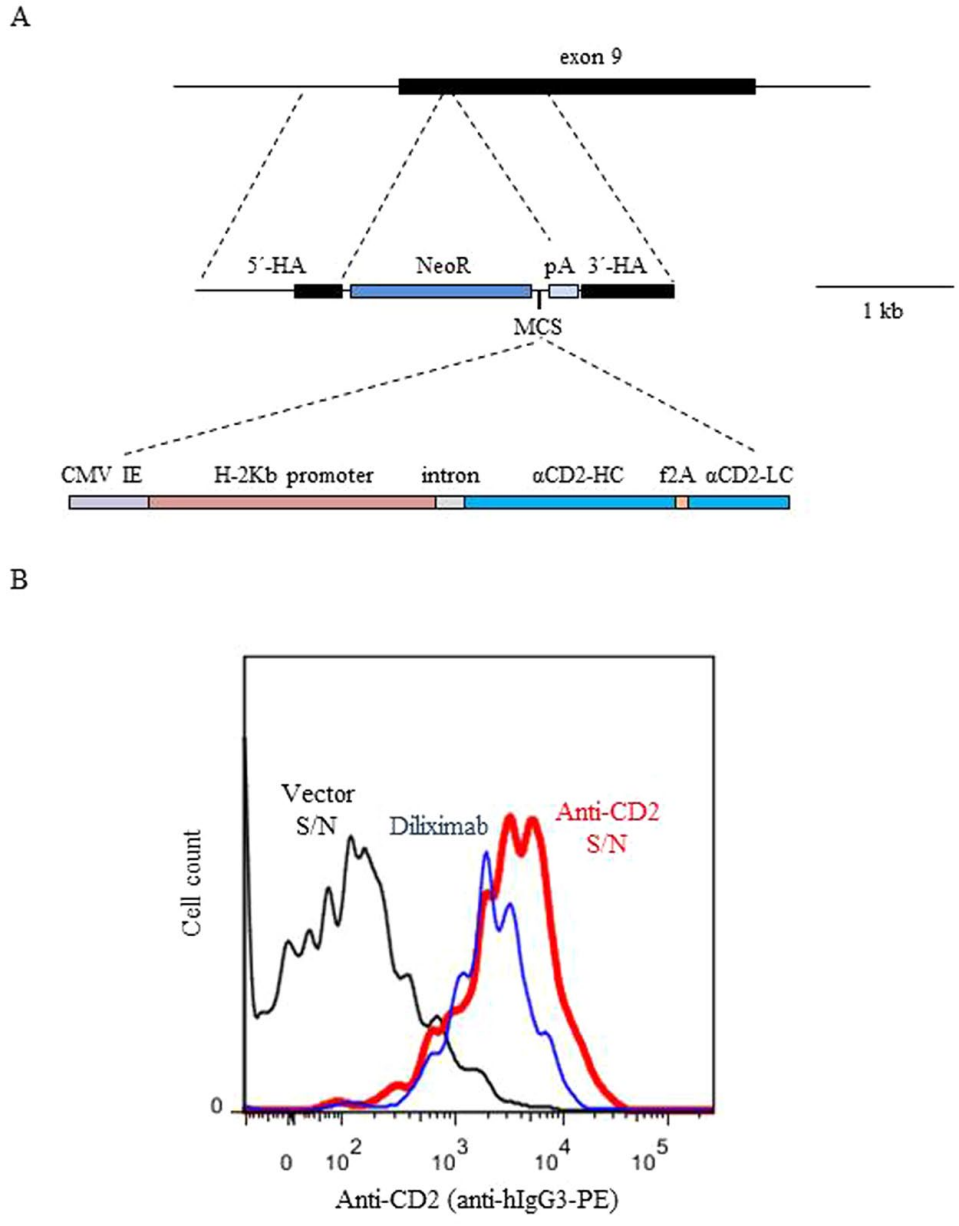
Validation of the anti-CD2 mAb *GGTA1* targeting construct. (**A**) Top, *GGTA1* genomic structure. Middle, knock-in backbone showing 5′ and 3′ homology arms (5′-HA and 3′-HA), neomycin resistance cassette (NeoR), multiple cloning site (MCS), and polyadenylation signal (pA). Into this was cloned (bottom) the CMV immediate early enhancer (CMV IE), mouse H-2K^b^ promoter, intron, and coding regions for the heavy and light chains of anti-CD2 mAb diliximab (αCD2-HC and αCD2-LC) linked by a furin cleavage site-F2A ribosome skip signal (f2A). The isotype of diliximab is human IgG3. (**B**) Detection of anti-CD2 mAb diliximab secreted by stably transfected COS-7 cells. Human leukocytes were incubated with culture supernatant, and mAb binding to CD3^+^ T cells was detected with anti-human IgG3. Red line, supernatant (S/N) from COS-7 cells transfected with the anti-CD2 knock-in construct; blue line, positive control (62.5 ng/ml purified diliximab); black line, supernatant from vector-transfected cells.

We designed a 3.6 kb *GGTA1* knock-in ‘backbone’ containing a 1,060 bp 5′ homology arm (including part of intron 8 and exon 9), a neomycin resistance cassette flanked by LoxP sites, a multiple cloning site (MCS) followed by a polyadenylation signal, and a 740 bp 3′ homology arm (exon 9) (Fig. 2A). Into the MCS, we cloned stepwise the CMV immediate early enhancer, the mouse H-2K^b^ promoter with a hybrid intron^2^, and the coding regions for the heavy and light chains of the anti-CD2 monoclonal antibody diliximab^5^ (Fig. 2A). Mouse IgG1 Fc has been substituted with human IgG3 Fc in this antibody^5^. The heavy and light chain coding regions were linked by a short sequence encoding a furin cleavage site fused to the F2A ribosome skip signal^17^, which together promote efficient mAb production *in vivo* ^18^. The sequence of the donor DNA template is shown in Suppl. Fig. 3.

To test the correct processing and secretion of the mAb, the knock-in construct (Fig. 2A) was stably transfected into COS-7 cells with neomycin selection, and the culture supernatant was harvested after the cells reached confluence. Human leukocytes were incubated first with culture supernatant and then with labelled anti-human IgG3, and analyzed by flow cytometry. The supernatant of cells transfected with the knock-in construct, but not that of cells transfected with vector alone, contained antibody that bound specifically to CD3^+^ human T cells (Fig. 2B), indicating secretion of diliximab.

### Generation and characterization of anti-CD2 knock-in pig fetal fibroblasts

**Figure 3:**
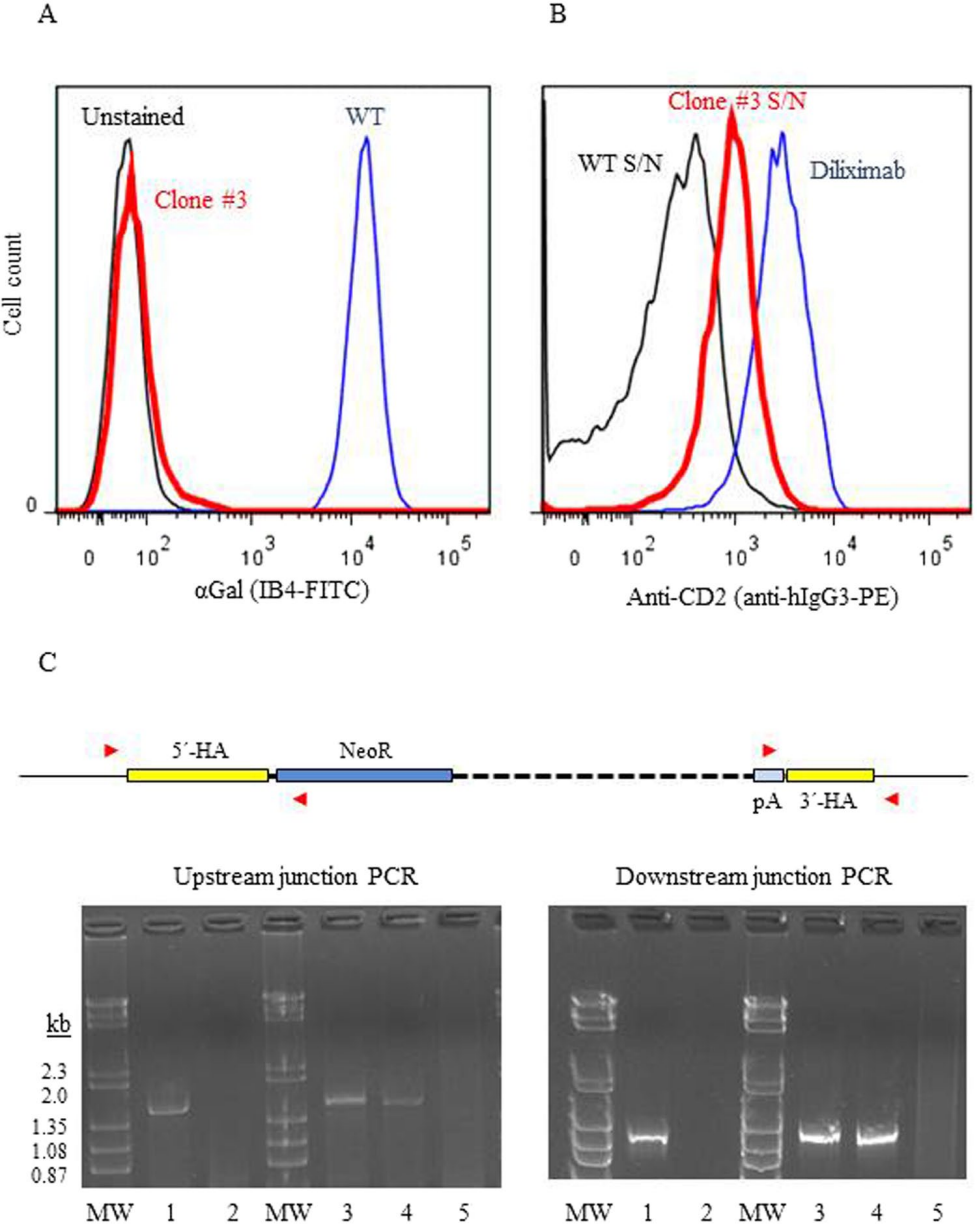
Analysis of pig fetal fibroblast anti-CD2 knock-in clone #3. (**A**) Absence of αGal expression by clone #3 (red line); blue line, WT fibroblasts; black line, unstained WT fibroblasts. (**B**) Presence of anti-CD2 mAb diliximab in the supernatant of clone #3 (red line) detected as described in the legend to Fig. 2; blue line, positive control (20 ng/ml purified diliximab); black line, supernatant from WT fibroblasts. (**C**) PCR analysis to confirm correct targeting in clone #3 and in two piglets generated from clone #3 by somatic cell nuclear transfer. The schematic diagram (top) shows the expected genomic configuration for integration of the knock-in construct in *GGTA1*; the upstream and downstream homology arms (HA) are shown in yellow. Two primer pairs (red arrowheads; 5′ = UKI-F3/UKI-R2; 3′ = 117-F/1123-R), each with one primer outside and one primer within the targeting construct, were used with genomic DNA isolated from clone #3 fibroblasts (lanes 1), WT fibroblasts (lanes 2), two cloned piglets (lanes 3 and 4), and one WT piglet (lanes 5). Clone #3 and both cloned piglets generated upstream and downstream products of the expected size (1506 bp, lanes 1, 3 and 4, left hand gel; and 947 bp, lanes 1, 3 and 4, right hand gel, respectively), which were confirmed by sequencing. MW, molecular weight markers (λ/*Hin*dIII + θX174/*Hae*III).

WT male pig fetal fibroblasts were co-transfected with the anti-CD2 knock-in construct (Fig. 2A) and expression vectors for *Fok*I-dCas9, GT-3 and GT-4. Neomycin selection was applied three days after transfection, and 12 neomycin-resistant clones were picked three weeks later. 3/12 clones were successfully expanded, and one of these (clone #3) tested αGal-negative by flow cytometry (Fig. 3A). Screening of supernatant from the culture of clone #3 on human T cells demonstrated the presence of anti-CD2 mAb diliximab (Fig. 3B).

PCR and sequencing of clone #3 genomic DNA confirmed that the knock-in construct was integrated correctly within exon 9 of *GGTA1* (Fig. 3C and Suppl. Fig. 4). PCR with primers designed to detect the *Fok*I-dCas9 and GT-3/GT-4 expression vectors (Suppl. Table 2) failed to generate products, indicating that these vectors had not integrated into the porcine genome. To determine the status of the second *GGTA1* allele, the target region was amplified from clone #3 genomic DNA using the primers shown in Fig. 1A and sequenced, revealing a deletion of 43 bp overlapping the recognition sites of GT-3 and GT-4 (Suppl. Fig. 5B). Together these data indicated that one allele of *GGTA1* was disrupted by homology-directed repair (HDR) using the knock-in construct as template, while the second allele was inactivated by a deletion resulting from non-homologous end-joining (NHEJ).

To demonstrate the versatility of the system, the 2.4 kb coding region in the anti-CD2 knock-in construct (Fig. 2A) was replaced by a 3.7 kb cDNA for the human anticoagulant protein thrombomodulin (hTBM)^19^. Using the transfection and selection procedure described above, 240 neomycin-resistant pig fetal fibroblast clones were isolated, of which 11 were both αGal-negative and expressed hTBM on the cell surface (Suppl. Fig. 6). One clone (#107) was analyzed further by PCR and sequencing, revealing precise knock-in of the 8.4 kb hTBM transgene into one allele of *GGTA1* and a 14 bp deletion in the second allele (Suppl. Fig. 5C).

### Cloning and analysis of anti-CD2 knock-in pigs

**Figure 4:**
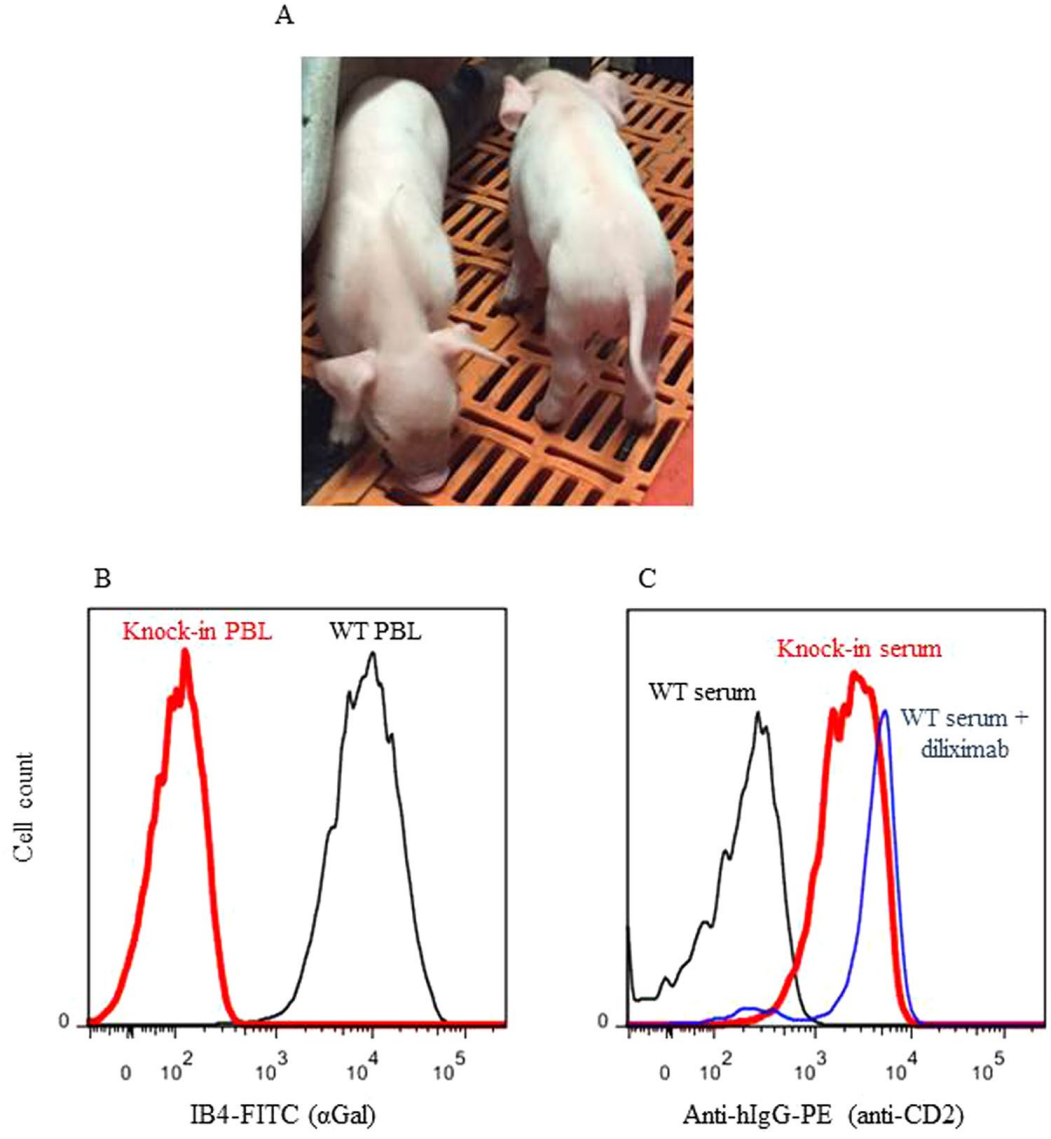
Anti-CD2 knock-in pigs. (**A**) Healthy appearance of the pigs. (**B**) Absence of αGal expression on peripheral blood leukocytes (PBL) (red line); blue line, WT leukocytes; black line, unstained. (**C**) Presence of anti-CD2 mAb diliximab in the serum (diluted 1:50) of a knock-in pig (red line), detected as described in the legend to Fig. 2; blue line, positive control (WT pig serum spiked with 200 ng/ml purified diliximab); black line, WT pig serum.

Anti-CD2 mAb knock-in clone #3 was used to generate pigs by somatic cell nuclear transfer (SCNT). Nine transfers of 90–130 reconstructed one-cell embryos were performed, resulting in two pregnancies. One pregnancy was carried to term and produced four live born male piglets, of which two died of natural causes shortly after birth. The surviving piglets were healthy and developed normally (Fig. 4A). PCR of genomic DNA prepared from ear biopsies confirmed correct integration of the transgene in *GGTA1* (Fig. 3C). Flow cytometric analysis demonstrated the absence of αGal expression on peripheral blood leukocytes (Fig. 4B), and the presence of anti-CD2 mAb diliximab in the sera of the pigs as detected by IgG3 (Fig. 4C).

## Discussion

We describe here an efficient method for knocking relatively large transgenes (up to 8.4 kb) into the *GGTA1* locus in pig cells using the high-fidelity *Fok*I-dCas9 system. Our initial experiment showed that *Fok*I-dCas9 used with two guide RNAs was capable of rapidly generating a sizeable proportion of biallelic *GGTA1* KO pig cells, even in the absence of selection. The efficiency of *GGTA1* KO (GTKO) using *Fok*I-dCas9 was approximately two-thirds that of WT Cas9, similar to what has been previously reported for other loci^10^. By including a targeting construct containing a transgene and an antibiotic selection marker, knock-in clones expressing the transgene could be isolated within one month of transfection. The desired genotype was obtained at a sufficient frequency that it was not necessary to enrich for KO pig cells, which has previously been done by flow cytometric cell sorting^6^ or magnetic bead selection^7, 20^. The successful expression of two knocked-in constructs (anti-CD2 and hTBM) confirmed that exon 9 of *GGTA1* is a permissive site for transcription of integrated transgenes^21^. Molecular analysis indicated knock-in of the transgenes by HDR at only one allele of *GGTA1*, with inactivation of the second allele by NHEJ. This is consistent with the observation that NHEJ is preferred over HDR at *Fok*I-dCas9-mediated double-stranded DNA breaks^22^.

Two healthy male pigs were generated from the anti-CD2 mAb knock-in cells by SCNT. While the technical difficulty of SCNT might be viewed as a limitation of the method, the recent generation of GTKO pigs by intracytoplasmic microinjection of CRISPR/Cas9 DNA into zygotes^23^ suggests that a simpler alternative may be available. To our knowledge, the 7.1 kb anti-CD2 transgene is the largest CRISPR-mediated knock-in that has been taken to the stage of producing viable pigs. Both pigs were GTKO and expressed anti-CD2 in their serum without apparent detrimental effects, presumably because the mAb is specific for human and primate CD2^4, 5^ and is not expected to deplete pig T cells. The knock-in pigs will be bred with GTKO-CD55-CD59-HT pigs to generate donors for our pig-to-baboon neonatal islet xenotransplantation model^24^. This will allow us to test the hypothesis that xenograft-specified expression of diliximab (that recognizes both human and primate CD2) will prolong xenograft survival by locally depleting infiltrating recipient T cells. Although we did not measure the frequency of off-target mutations, there is already ample evidence to support the greater specificity of *Fok*I-dCas9^10, 25^ and the closely related fdCas9^11^ compared to WT Cas9. Furthermore, breeding of the knock-in pigs is expected to dilute detrimental off-target mutations, if any.

The *GGTA1* knock-in backbone was designed for versatility by incorporating a multiple cloning site to enable simple interchange of transgene regulatory elements and/or coding regions. The neomycin resistance cassette is flanked by LoxP sites and can be excised using Cre recombinase if desired. In addition, the backbone can be modified to incorporate a different antibiotic selection marker, potentially enabling simultaneous knock-in of different transgenes into the two alleles of *GGTA1*. The relative ease with which the 8.4 kb hTBM transgene was knocked into the pig genome suggests that the upper size limit for HDR using relatively short homology arms (1.06 and 0.74 kb) has not been reached. We therefore envisage that additional genes can be incorporated into the knock-in construct, either as separate transcriptional units or by using 2A for multi-cistronic expression.

## Methods

All experiments with animals and human samples were performed in accordance with relevant guidelines and regulations. Genetically engineered pigs were generated and used with the approval of the Animal Ethics Committee of the University of Adelaide (approval number M-2012-084B), in accordance with the *Australian Code of Practice for the Care and Use of Animals for Scientific Purposes* (National Health and Medical Research Council, 2013). Human blood was drawn from healthy volunteers with informed consent and with the approval of the St Vincent’s Hospital Melbourne Human Research Ethics Committee.

### Reagents

Unless otherwise specified, chemicals were obtained from Sigma Aldrich (Sydney, Australia), and cell culture and molecular biology reagents were obtained from Thermo Fisher Scientific (Scoresby, Australia).

### Preparation of knock-in constructs

The 3.58 kb *GGTA1* knock-in backbone, including flanking *Not*I sites, was synthesized by GenScript (Piscataway, NJ) and cloned in the vector pUC57. It contained a MCS including sites (5′ to 3′) for *Fse*I, *Asc*I and *Eco*RI. The anti-CD2 mAb and hTBM knock-in constructs were prepared in three steps. First, the 0.66 kb CMV immediate early enhancer was amplified by PCR of the vector pCI-Neo (Promega, Alexandria, Australia) using *Fse*I-containing primers, and cloned into the *Fse*I site of the knock-in backbone. Second, a 2.2 kb PCR product containing a 2.0-kb mouse H-2K^b^ promoter and a 0.2 kb hybrid intron was amplified from a previously described transgenic construct^26^ using *Asc*I-containing primers, and cloned into the *Asc*I site. Finally, an *Eco*RI fragment encoding either the anti-CD2 mAb diliximab^5^ (2.4 kb) or hTBM^19^ (3.7 kb) was cloned into the *EcoRI* site. The anti-CD2 *EcoRI* fragment consisted of the coding regions for the heavy and light chains linked by a furin cleavage site/F2A ribosome skip signal, and was synthesized by GenScript. All constructs were fully sequenced. *Not*I, *Fse*I and *Asc*I were obtained from New England Biolabs (Genesearch, Arundel, Australia).

### Tissue culture

Cells were grown in 5% CO_2_ at 37 °C. WT male pig fetal fibroblasts (Large White/Landrace) were grown in 0.01% gelatin-coated flasks in 50% Dulbecco’s Modified Eagle’s Medium (DMEM), 50% M199, 10% heat-inactivated fetal calf serum (FCS), penicillin/streptomycin and 5ng/ml bFGF, and used at passage 7. COS-7 cells were cultured in DMEM with 10% heat-inactivated FCS.

### Transfection

Transfection of WT porcine fetal fibroblasts was performed using an Amaxa 4-D Nucleofector using the P3 Nucleofector Kit and the EN150 program (Lonza, Mount Waverley, Australia). 10^6^ cells were transfected with a total of 5 μg of DNA, comprising equal amounts of the expression vectors for *Fok*I-dCas9 (plasmid #52970, Addgene, Cambridge, MA) and the single guide RNAs (GenScript), plus/minus the knock-in construct (excised from the pUC57 vector using *Not*I). Three days after transfection, the cells were either harvested for analysis or neomycin (G418, 800 μg /ml) was added to select stable transfectants.

Transfection of COS-7 cells was performed using a GenePulser electroporator (Bio-Rad, Gladesville, Australia). 5 × 10^6^ cells were transfected with 5 μg of the anti-CD2 mAb knock-in construct at 250 volts and 950 μFd. Three days after transfection, G418 (800 μg/ml) was added to select stable transfectants. When the transfected cells reached confluence, the culture supernatant was collected for detection of anti-CD2 mAb diliximab as described below.

### Flow cytometry

For analysis of cell surface expression of αGal or hTBM, cells were stained with FITC-conjugated *Griffonia simplicifolia* I-B4 lectin (IB4) or anti-hTBM clone IA4 (kind gift of Dr Phillip Bird, Monash University, Melbourne, Australia), respectively, and analyzed on a FACSCanto II (Becton Dickinson, North Ryde, Australia) as described^17, 26^. Secreted anti-CD2 mAb diliximab was detected as follows using human T cells as targets. Leukocytes were isolated from human blood using Ficoll Paque Plus gradients (GE Healthcare, Murarrie, Australia). 10^5^ leukocytes were incubated for 1 hr with culture supernatant or serum, or purified diliximab^5^ as positive control. After washing, the cells were incubated with goat anti-human IgG-biotin (Thermo Fisher Scientific, catalog #12-4998-82) and anti-human CD3-FITC (BD Biosciences, North Ryde, Australia; catalog #555332) for 30 minutes. The cells were analyzed on the FACSCanto II using CD3^+^ gating of T cells.

### Somatic cell nuclear transfer

Porcine oocyte collection and *in vitro* maturation in BOMED maturation medium were performed as described^27, 28^. Cell culture before nuclear transfer was performed as described^27^ except that cells were dissociated by incubation for 5 min in TrypLE. Nuclear transfer was performed using the fusion before activation protocol as described^27^.

## Electronic supplementary material

Supplementary information
