# Supplementary material for: Targeted insertion of an anti-CD2 monoclonal antibody transgene into the GGTA1 locus in pigs using FokI-dCas9
Source: Sci Rep. 2017 Aug 16;7:8383. doi: 10.1038/s41598-017-09030-6 (PMC5559588; doi:10.1038/s41598-017-09030-6)
Supplement: Supplementary file 1 — Supplementary information [file 41598_2017_9030_MOESM1_ESM.pdf]

Supplementary Information

Targeted insertion of an anti-CD2 monoclonal antibody transgene into the *GGTA1* locus in pigs using *Fok*I-dCas9

Mark B. Nottle, Evelyn Salvaris, Nella Fisicaro, Stephen McIlfatrick, Ivan Vassiliev, Wayne J. Hawthorne, Philip J. O'Connell, Jamie L. Brady, Andrew M. Lew, Peter J. Cowan

Supplementary Figures 1-6

Supplementary Tables 1 and 2

Supplementary Figure 1

Targeting of *GGTA1* in WT pig fetal fibroblasts using Cas9

WT fibroblasts were co-transfected with expression vectors for Cas9 (pGS-CMV-hCas9, GenScript) and guide RNA GT-6. Flow cytometric analysis of the cells at day 3 (red line) demonstrated that ~30% were αGal-negative; blue line, control vector-transfected WT cells; black line, unstained WT cells.

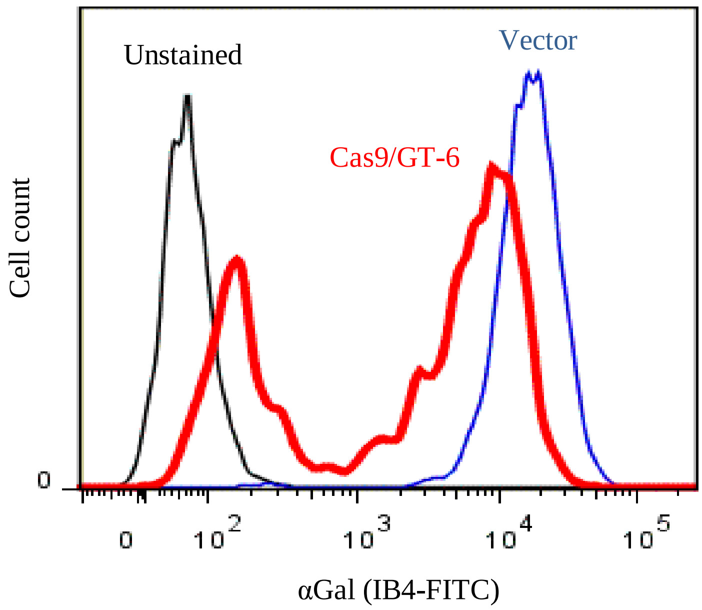

Supplementary Figure 2

Surveyor nuclease assay to detect indels in *GGTA1* in pig cells

WT fibroblasts were transfected with the combinations of expression vectors shown. pGS-CMV-hCas9 was from GenScript, and pCas9-GFP was from Addgene (plasmid #44719). Three days after transfection, genomic DNA was isolated and a 970 bp region of *GGTA1* encompassing the target site was amplified by PCR using primers SN-F2 and SN-R2. The 970 bp PCR product was analysed using the Surveyor Mutation Detection kit (Transgenomic, Omaha, NE). Bands of approximately 592 bp and 378 bp (arrowheads) indicate cleavage at the expected target site. *Fok*I-dCas9 generated mutations with both pairs of guide RNAs, albeit with a lower efficiency than Cas9 with a single guide RNA. Left lane: molecular weight markers (λ/*Hin*dIII + φX174/*Hae*III).

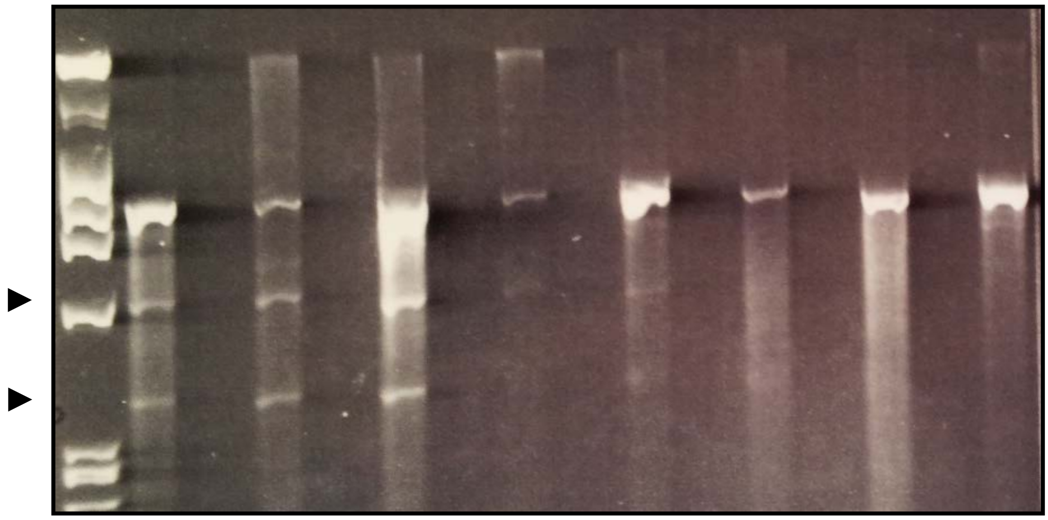

pGS-CMV-hCas9	+	+	-	-	-	-	-	-
pCas9-GFP	-	-	+	+	-	-	+	-
*Fok*I-dCas9	-	-	-	-	+	+	-	-
gRNA	5	6	5	6	1+2	3+4	-	-

Supplementary Figure 3

Sequence of donor DNA template for the generation of anti-CD2 mAb knock-in pig fibroblasts

GCGGCCGCTGCAACTGCAATTAGACCCCTAGTCTGGGAACCTCCATATGCTGCGGATGTGGCCCTAAA

AAGACAAAAAATAAAAATTAAAAATAAGTGCATCACATCAACACTTTGCACACCTTAAACTTACACAA

TGATATATGTCAGTTATATCTGAATAATGCAGGAAAAAAGGAAGAACTAGCATCTGAACTGATTGATA

AGGACAAGGGGATAAAGACTAATTAAGGCAGATGGTAATGAGTTATCACCTTCATTACAGTTAAAGCT

GGTGTTGGAGTATGTGGCTTATAACTGCAGGCCCTGGGCTTCCAAATCTTGAACCACAGAATTAGAAA

GACTTAGGCATCCAGGCACATACAGCACAAGTAACTGCAGGCCTCTGGATGTGGGAGCAGGGCCTGTG

CCACTAAGGTACCACTGCAAGGCCCAGAGCAGCTGAAAACACATGTTCTCTCTGCCTGGTTGGCTTCC

AAGAGTGAGAGAGGAAGGAGCAGGGCTGAGCATGCCCAGCCACCCTGCCAGAATCACCAGTCAGGTAA

GCCACTCCACCTCCCCAAAGCTGAATGACTGAATGGTGGAGAGTAGCTGGGAATGTTACAGCAACAGA

CGTCTCTCATCCAGGATGGGGAAAAATCATTCCTTTCCTAAACTGCAAAATACAGACTAGATGATAAT

AGCATATTGTCTCCTCTAGAAATCCCAGAGGTTACATTTACCCCATTCTTCTTTATTTCAGATACATT

GAGCATTACTTGGAGGAGTTCTTAATATCTGCAAATACATACTTCATGGTTGGCCACAAAGTCATCTT

TTACATCATGGTGGATGATATCTCCAGGATGCCTTTGATAGAGCTGGGTCCTCTGCGTTCCTTTAAAG

TGTTTGAGATCAAGTCCGAGAAGAGGTGGCAAGACATCAGCATGATGCGCATGAAGACCATCGGGGAG

CACATCCTGGCCCACATCCAGCACGAGGTGGACTTCCTCTTCTGCATGGACGTGGATCAGGTCTTCCA

AAACAACTTTGGGGTGGAGACCCTGGGCCAGTCGGTGGCTCAGCTAAGATCTATAACTTCGTATAGCA

TACATTATACGAAGTTATAATTCTACCGGGTAGGGGAGGCGCTTTTCCCAAGGCAGTCTGGAGCATGC

GCTTTAGCAGCCCCGCTGGCACTTGGCGCTACACAAGTGGCCTCTGGCCTCGCACACATTCCACATCC

ACCGGTAGCGCCAACCGGCTCCGTTCTTTGGTGGCCCCTTCGCGCCACCTTCTACTCCTCCCCTAGTC

AGGAAGTTCCCCCCCGCCCCGCAGCTCGCGTCGTGCAGGACGTGACAAATGGAAGTAGCACGTCTCAC

TAGTCTCGTGCAGATGGACAGCACCGCTGAGCAATGGAAGCGGGTAGGCCTTTGGGGCAGCGGCCAAT

AGCAGCTTTGCTCCTTCGCTTTCTGGGCTCAGAGGCTGGGAAGGGGTGGGTCCGGGGGCGGGCTCAGG

GGCGGGCTCAGGGGCGGGGCGGGCGCGAAGGTCCTCCGGAGCCCGGCATTCTCGCACGCTTCAAAAGC

GCACGTCTGCCGCGCTGTTCTCCTCTTCCTCATCTCCGGGCCTTTCGGCTGCAGCCACCATGATTGAA

CAAGATGGATTGCACGCAGGTTCTCCGGCCGCTTGGGTGGAGAGGCTATTCGGCTATGACTGGGCACA

ACAGACAATCGGCTGCTCTGATGCCGCCGTGTTCCGGCTGTCAGCGCAGGGGCGCCCGGTTCTTTTTG

TCAAGACCGACCTGTCCGGTGCCCTGAATGAACTGCAGGACGAGGCAGCGCGGCTATCGTGGCTGGCC

ACGACGGGCGTTCCTTGCGCAGCTGTGCTCGACGTTGTCACTGAAGCGGGAAGGGACTGGCTGCTATT

GGGCGAAGTGCCGGGGCAGGATCTCCTGTCATCTCACCTTGCTCCTGCCGAGAAAGTATCCATCATGG

CTGATGCAATGCGGCGGCTGCATACGCTTGATCCGGCTACCTGCCCATTCGACCACCAAGCGAAACAT

CGCATCGAGCGAGCACGTACTCGGATGGAAGCCGGTCTTGTCGATCAGGATGATCTGGACGAAGAGCA

TCAGGGGCTCGCGCCAGCCGAACTGTTCGCCAGGCTCAAGGCGCGCATGCCCGACGGCGAGGATCTCG

TCGTGACCCATGGCGATGCCTGCTTGCCGAATATCATGGTGGAAAATGGCCGCTTTTCTGGATTCATC

GACTGTGGCCGGCTGGGTGTGGCGGACCGCTATCAGGACATAGCGTTGGCTACCCGTGATATTGCTGA

AGAGCTTGGCGGCGAATGGGCTGACCGCTTCCTCGTGCTTTACGGTATCGCCGCTCCCGATTCGCAGC

GCATCGCCTTCTATCGCCTTCTTGACGAGTTCTTCTGAGCGGGACTCTGGGGTTCGAAATGACCGACC

AAGCGACGCCCAACCTGCCATCACGATGGCCGCAATAAAATATCTTTATTTTCATTACATCTGTGTGT

TGGTTTTTTGTGTATAACTTCGTATAGCATACATTATACGAAGTTATAGATCTGCTCGAGGCCGGCCT

TTCAATATTGGCCATTAGCCATATTATTCATTGGTTATATAGCATAAATCAATATTGGCTATTGGCCA

TTGCATACGTTGTATCTATATCATAATATGTACATTTATATTGGCTCATGTCCAATATGACCGCCATG

TTGGCATTGATTATTGACTAGTTATTAATAGTAATCAATTACGGGGTCATTAGTTCATAGCCCATATA

TGGAGTTCCGCGTTACATAACTTACGGTAAATGGCCCGCCTGGCTGACCGCCCAACGACCCCCGCCCA

TTGACGTCAATAATGACGTATGTTCCCATAGTAACGCCAATAGGGACTTTCCATTGACGTCAATGGGT

GGAGTATTTACGGTAAACTGCCCACTTGGCAGTACATCAAGTGTATCATATGCCAAGTCCGCCCCCTA

TTGACGTCAATGACGGTAAATGGCCCGCCTGGCATTATGCCCAGTACATGACCTTACGGGACTTTACT

ACTTGGCAGTACATCTACGTATTAGTCATCGCTATTACCATGGTGATGCGGTTTTGGCAGTACACCAA

TGGGCGTGGATAGCGGTTTGACTCACGGGGATTTCCAAGTCTCCACCCCATTGACGTCAATGGGAGTT

TGTTTTGGCACCAAAATCAACGGGACTTTCCAAAATGTCGTAACAACTGGCCGGCCTTAATTAAGGCG

CGCCTCATAGACTCATGTGTTTGAAAGCTTGGCCCACAAGGAGTGGCAATGTTAGGAGTGTGACTGTG

TTAGAGGAAGTGTGTCACTTTGAGGTTGGCCTTTGAGGTCTCCTATGCTCATGAGCTGCCCAGTGTGG

AATGGAAGCCTCTTCCTGGCTGCATTTGGATCAAGATGTAGAAGTCTCCCCTTCTCCAGCACCATGTC

TACCTGCACACTGGCATGCTTCCTGTCATGATGGACTAAATCCCTGAAACTGTAAGCCAGACCCTATT

AAATGTCTCCCTTTAGAAGGAATGCCTTGGTCATGGTGTTTCTTCACAGCAACAAAACCAAAACTAAC

ACACCCAGCACTTAGGATGCAAAGGCAGATGTATCTCTGTGAGTTCAAGGGCAGCCTGATCTACATAA

TGAATTACAGAATAGCCAGGGCTGTGCAGATAGACCTTGTCTAAACAAGCAAACAAACAAAACCCATA

GTAAAATAAGAAGAAAAGTAATGGAGACAGATCCTTTGTGTAGCTGCCTAACCCCAACAGCATCCCTA

GGTTGATCAGGAAGAAACCTAAGAGCATCAAGTCCCCATGGAAAATGCTCACAACATCTAATAAGAGG

GGTTCAGGGAGACAGGGTCTGCTGAATCCTGGTGAGGCTGGGGGGCTGCTGTGGGGATCTGCATGGGG

AGGTGACTGGAGTTCACACCTGTGGTCACACGCATTCATTGCTCTGTGTGATGAGTGACTCTCTTCAG

GAGTCCCCACCCATCTATGCTCTTCTTTCCACTTCACTTCTGTGACTGATTTGAAAAGATCCACAGAA

TCATGAAATGGGTAAATTTAATCTTTCCCCACACTACTGATCAGGGATGAAACCTCACATCACAGTGT

GTGCTCTCTGGCATGAGAATCATCTTTCTCCCAGTGTCCACACTGCACAGGCCTGAGGAACTCTGGGG

CCAACTCAATTATCAGAACCCGTGTCCCACACAGCTGTGACATTCATGTCCCTCACCTCATCAGCTCA

AAAGTGTGAGTGAAGGTCAGGGAGGGAGGGAGAGGGTCAGAGATTACATAACTTCTACCACACTAGCA

TCCTTATTCTGTGTTATAGTTGGTGACAGCTCCTCTCCTACTGTGATTGAGTCACTAAAGCAACTGCA

CCATGGAAGTGCAGACACAGAGAAGTCCAGTGGAGACAGGGATCAGTGCCCTGCTGTCTGTGGTCTGC

ACGGGGTCTCTCTTCAGTGGACAAGGGGGTCTCCTGTGCTGAGACAATGTCCCAGATCCACAGAGACA

AACTCAGGACTCAGAATGAAGATCCTTGTTTCAAATACACACACACACACATACACACACACACACAC

ACACACACACACACACACACACACCTGCAGCCACAGACTTTTCATCTAGGAATTAACACAAGGAATCT

GTGTCTCAGCACAGGGCTGAGAAGACAGATCCTGAGGGGAGAGGCAAAGTCTACACTTGACAGATGAG

AGTCCTGCACTCAGGCTTGGCAGTGTGAGCCGCCCATTGCAGGTGAACAGAGCCTGGTCTCTGTGGGG

TCCCTGTGGGGCTTGCAGCCCAGCGCCTTGACTTTAAGGAAAAGCCTCTCTCTCCACTGCATCCCTAA

GCGCTTGTGTCGCCATTGTATTCCCGGAAGAGGCTTTTCTTCTAGAAGACTCCAGGGTCTGACTTCTG

AAGAGAAGAAGAAAGAGGAAGAGTGGAAGAGAGGACACAGAGAGTCTGGCCTGCGGGTCTCTCCTGGT

GTTTTGAGAGTTTCTGGATCAGAACTCGGAGACGACAGCACAGGGTTCAGGCAAAGTCTTAGTCGCCA

GGCAGTGAGGTCAGGGGTGGGGAAGCCCAGGGCTGGGGATTCCCCATCTCCACAGTTTCACTTCTGCA

CCTAACCTGGGTCAGGTCCTTCTGTCCGGACACTGTTGACGCGCAGTCAGCTCTTACCCCCATTGGGT

GGCGCGATCACCAAGAACCAATCAGTGTCGCCGCGGACGCTGGATATAAAGTCCACGCAGCCCGCAGA

ACTCAGAAGTCGATCAAGCTTATCGATTGGGGTGAGTACTCCCTCTCAAAAGCGGGCATGACTTCTGC

GCTAAGATTGTCAGTTTCCAAAAACGAGGAGGATTTGATATTCACCTGGCCCGCGGTGATGCCTTTGA

GGGTGGCCGCGTCCATCTGGTCAGAAAAGACAATCTTTTTGTTGTCAAGCTTGAGGTGTGGCAGGCTT

GAGATCTGACCATACACTTGAATGACAATGACATCCACTTTGCCTTTCTCTCCACAGGCGCGCCGGAT

CCGAATTC-coding region for anti-CD2 monoclonal antibody (2,367 bp)-

GAATTCCGACTGTGCCTTCTAGTTGCCAGCCATCTGTTGTTTGCCCCTCCCCCGTGCCTTCCTTGACC

CTGGAAGGTGCCACTCCCACTGTCCTTTCCTAATAAAATGAGGAAATTGCATCGCATTGTCTGAGTAG

GTGTCATTCTATTCTGGGGGGTGGGGTGGGGCAGGACAGCAAGGGGGAGGATTGGGAAGACAATAGCA

GGCATGCTGGGGATGCGGTGGGCTCTATGGGTGGTACAAGGCACATCCTGACGAGTTCACCTACGAGA

GGCGGAAGGAGTCCGCAGCCTACATTCCGTTTGGCCAGGGGGATTTTTATTACCACGCAGCCATTTTT

GGGGGAACACCCACTCAGGTTCTAAACATCACTCAGGAGTGCTTCAAGGGAATCCTCCAGGACAAGGA

AAATGACATAGAAGCCGAGTGGCATGATGAAAGCCATCTAAACAAGTATTTCCTTCTCAACAAACCCA

CTAAAATCTTATCCCCAGAATACTGCTGGGATTATCATATAGGCATGTCTGTGGATATTAGGATTGTC

AAGATAGCTTGGCAGAGAAAAGAGTATAATTTGGTTAGAAATAACATCTGACTTTAAATTGTGCCAGC

AGTTTTCTGAATTTGAAAGAGTATTACTCTGGCTACTTCCTCAGAGAAGTAGCACTTAATTTTAACTT

TTAAAAAAATACTAACAAAATACCAACACAGTAAGTACATATTATTCTTCCTTGCAACTTTGAGCCTT

GTCAAATGGGAGAATGACTCTGTGGTAATCAGATGTAAATTCCCAATGATTTCTTATCTGTTCTGGGT

TGAGGGGGTATATACTATTAACTGAACCAAAAAAAAAATTGTCATAGGCAAAGAAAAAGTCAGAGACA

CTCTACATGTCATACTGGAGAAAAGTATGCAAAGGGAAGTGTTTGGCAACAAAATAAGATTGGGAGGG

GTCGTCCTCTTGATTTTAGCGGCCGC

Supplementary Figure 4

Sequencing chromatograms of the junction regions of the targeted *GGTA1* allele in anti-CD2 mAb knock-in clone #3

(**A**) Sequence of 5´ junction. Highlighted in yellow are (i) the recognition site for primer UKI-F3, which lies upstream of the targeting construct, and (ii) the first base of the 5´ homology arm in the targeting construct. (**B**) Sequence of 3´ junction. Highlighted in yellow are (i) the last base of the 3´ homology arm in the targeting construct, and (ii) the recognition site for primer 1123-R, which lies downstream of the targeting construct.

A

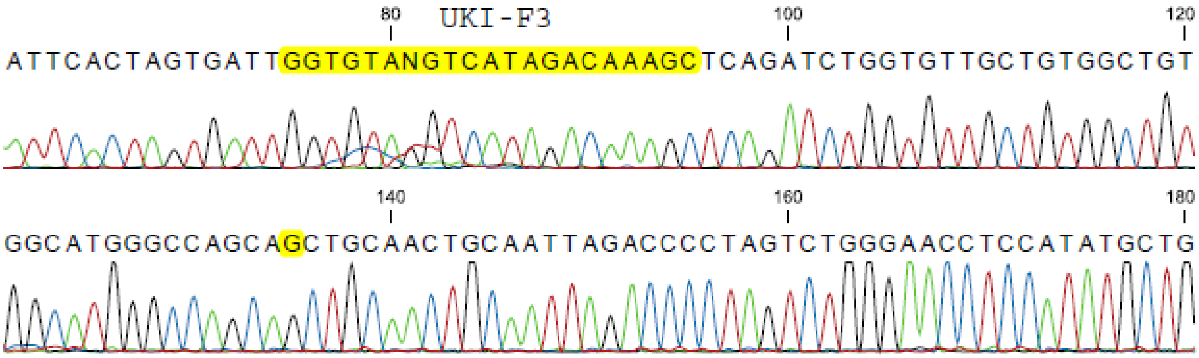

B

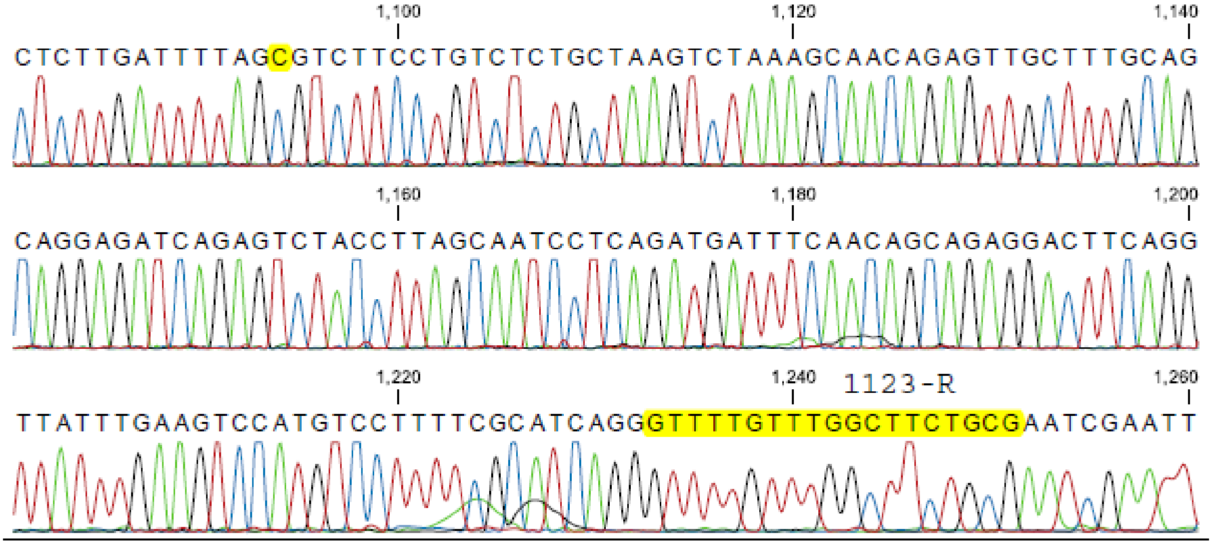

Supplementary Figure 5

Deletions detected within *GGTA1* in WT pig fibroblasts edited using *Fok*I-dCas9 with guide RNAs GT-3 and GT-4

The WT sequence (top) shows the recognition sites for GT-3 and GT-4 (underlined). (**A**) Clone isolated during the initial testing of GT-3 and GT-4, showing a 25 bp deletion. (**B**) Anti-CD2 knock-in clone #3, showing a 43 bp deletion. (**C**) hTBM knock-in clone #107, showing a 14 bp deletion.

```
WT  GTCGGTGGCTCAGCTACAGGCCTGGTGGTACAAGGCACATCCTGACGAGTTCACCTACGAGAGG
A   GTCGGTGGCTCAGCTACAGGCCTGGTGGT-------------------------CTACGAGAGG
B   GTCGGTGGCT-------------------------------------------CCTACGAGAGG
C   GTCGGTGGCTCAGCTACAGGCC--------------ACATCCTGACGAGTTCACCTACGAGAGG
```

Supplementary Figure 6

Absence of αGal and expression of hTBM in hTBM knock-in clone #107

(**A**) αGal and (**B**) hTBM staining. Blue line, clone #107; red line, vector-transfected WT cells; black line, unstained WT cells.

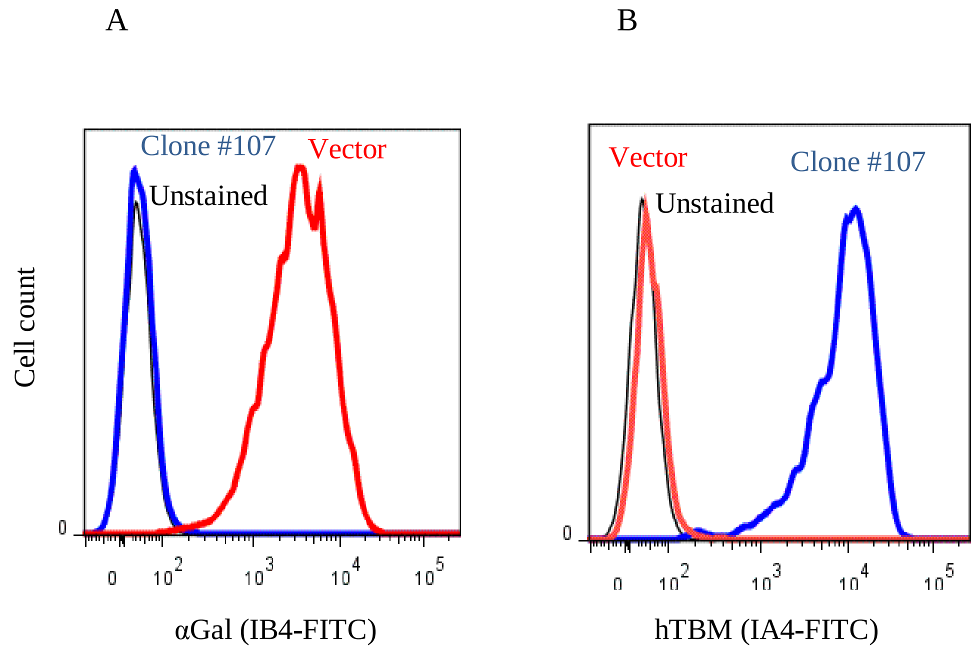

Supplementary Table 1

Single guide RNAs targeting *GGTA1*

**Name**	**Sequence 5´-3´**	**PAM**
GT-1	CTGAGCCACCGACTGGCCC	AGG
GT-2	TGACGAGTTCACCTACGAG	AGG
GT-3	CTGTAGCTGAGCCACCGAC	TGG
GT-4	CGAGTTCACCTACGAGAGG	CGG
GT-5	GGTGGCTCAGCTACAGGCC	TGG
GT-6	CAGTCGGTGGCTCAGCTAC	AGG

PAM: protospacer adjacent motif

Supplementary Table 2

PCR primers

**Target**	**Forward 5´-3´ (primer name)**	**Reverse 5´-3´ (primer name)**	**Size (bp)**
GGTA1	GCTGCAACTGCAATTAGACC (GTFS-F1)	GCTAAAATCAAGAGGACGACC (GTFS-R1)	1806
GGTA1	CCTCTAGAAATCCCAGAGGTTAC (SN-F2)	CCCTCAACCCAGAACAGATAAG (SN-R2)	970
Knock-in 5´ junction	GGTGTAGGTCATAGACAAAGC (UKI-F3)	AGAAAGCGAAGGAGCAAAG (UKI-R2)	1506
Knock-in 3´ junction	GAGGATTGGGAAGACAATAGC (117-F)	CGCAGAAGCCAAACAAAAC (1123-R)	947
*Fok*I-dCas9 vector	GAAGATGGATGGGACGGA (fCas9-F1)	CTTTTTGATGGCTGGCGA (fCas9-R1)	1063
gRNA vector (pGS-U6-gRNA)	ATCCTCATCCTGTCTCTT (gRNA-F2)	GTATGTTGTGTGGAATTGTG (gRNA-R1)	1518
